# Sex Differences in Cardiovascular Health Status and Long-Term Outcomes in a Primary Prevention Cohort

**DOI:** 10.1016/j.jacadv.2025.102108

**Published:** 2025-09-01

**Authors:** Maneesh Sud, Areeba Chaudhry, Feng Qui, Olivia Haldenby, Lucas C. Godoy, Peter C. Austin, Idan Roifman, Douglas Manuel, Dean T. Eurich, Harindra C. Wijeysundera, Mina Madan, Thao Huynh, Dennis T. Ko

**Affiliations:** a Schulich Heart Program, Sunnybrook Health Sciences Centre, University of Toronto, Toronto, Ontario, Canada; b ICES, Toronto, Ontario, Canada; c Temerty Faculty of Medicine, University of Toronto, Toronto, Ontario, Canada; d Peter Munk Cardiac Centre, University Health Network, Toronto, Ontario, Canada; e Institute of Health Policy Management, and Evaluation, University of Toronto, Toronto, Ontario, Canada; f Ottawa Hospital Research Institute, University of Ottawa, Ottawa, Ontario, Canada; g Department of Family Medicine, University of Ottawa, Ottawa, Ontario, Canada; h School of Public Health, University of Alberta, Edmonton, Alberta, Canada; i Department of Medicine, Faculty of Medicine and Health Sciences, McGill University, Montreal, Quebec, Canada; j Division of Cardiology, Department of Medicine, McGill University Health Centre, Montreal, Quebec, Canada

**Keywords:** cardiovascular health, outcomes, prevention, sex differences

## Abstract

**Background:**

Poor cardiovascular health quantified by 8 health behaviors and factors is associated with incident cardiovascular disease (CVD). However, it is not clear if this association differs between women and men.

**Objectives:**

The aim of the study was to determine whether the association between cardiovascular health status and incident CVD events differs by sex.

**Methods:**

Adults enrolled in the Ontario Health Study (March 2009-December 2017) with no prior CVD were included. Cardiovascular health was assessed based on the presence of ideal diet, sleep, physical activity, smoking, body habitus, blood glucose, blood cholesterol, and blood pressure. CVD events were ascertained to March 31, 2023. The association between ideal, intermediate, and poor health and outcomes was assessed using age-adjusted, cause-specific models, including interaction terms for sex.

**Results:**

The cohort consisted of 175,098 individuals (61.4% women, mean age 47 years). Compared to men, more women had ideal cardiovascular health (9.1% vs 4.6%), while fewer women had poor cardiovascular health (21.9% vs 30.5%). Compared to ideal cardiovascular health, the age-adjusted HR for intermediate cardiovascular health was 2.31 (95% CI: 1.72-3.11) in women, which was greater than the HR of 1.57 (95% CI: 1.16-2.11) in men. Similarly, the HR for poor cardiovascular health was 5.08 (95% CI:3.78-6.84) in women and nearly double the HR of 2.54 (95% CI: 1.89-3.41) in men (*P* < 0.01 for interaction).

**Conclusions:**

In this primary prevention cohort, as cardiovascular health status declined, the relative impact on the future rate of CVD was greater for women than for men. This supports sex-specific health promotion strategies to improve health status and outcomes.

Cardiovascular health can be quantified using 4 modifiable health behaviors (ie, sleep duration, smoking, physical activity, and diet) and 4 health factors (ie, obesity, blood cholesterol, blood glucose, and blood pressure).^1^^,^^2^ Several studies in the United States,^3^, ^4^, ^5^ China,^6^ and Europe^7^^,^^8^ have demonstrated that poor cardiovascular health is strongly associated with higher rates of incident CVD and death. Many individual health behaviors and factors (eg, smoking, sleep duration, blood pressure, glucose, and physical activity) confer a higher risk in women^9^, ^10^, ^11^, ^12^, ^13^ while others (eg, cholesterol and diet) confer a higher risk in men.^14^^,^^15^ It is therefore plausible that when considered collectively, the impact of health on long-term outcomes may vary by sex because of differences in biological and social determinants of health.^16^ Understanding the relative impact of cardiovascular health on outcomes by sex would support targeted interventions to improve equitable screening and health promotion to reduce the burden of CVD.^17^, ^18^, ^19^

Accordingly, our objective was to determine whether the association between cardiovascular health status and incident cardiovascular events differs by sex. We used the CANHEART (Cardiovascular Health in Ambulatory Care Research Team) health index, which was developed to examine the health of Canadians,^20^ and applied this index to a large, contemporary primary prevention cohort in Ontario, Canada.

## Methods

### Data sources

We utilized the OHS (Ontario Health Study), which is the largest prospectively collected longitudinal study in Canada, with over 200,000 participants residing in the province of Ontario.^21^ Participants were recruited through invitation e-mails and letters, advertising, primary care providers, other stakeholders, incentive programs, and friends and family referrals. Baseline health-related questionnaires were completed by participants at enrollment. Community-based study centers were created to collect blood, urine, and physical measurements from participants. A subset of over 40,000 participants provided blood samples, and 12,000 provided physical measurements. Anonymized linkages were performed between OHS and provincial administrative and laboratory databases by utilizing unique encoded identifiers and analyzed at ICES (formerly known as the Institute for Clinical Evaluative Sciences). These included: 1) the Canadian Institute for Health Information Discharge Abstract Database to determine previous as well as outcome hospitalization events;^22^ 2) the Ontario Registrar General Database for cause of death; 3) the Ontario Laboratories Information System for laboratory data;^22^^,^^23^ and 4) the Immigration, Refugees and Citizenship Canada’s Permanent Residents Database for recent immigration status current to 2023. This project has been approved by the Research Ethics Board at Sunnybrook Research Institute.

### Study cohort

We included Ontario residents with valid Health Insurance Numbers who completed the baseline questionnaire between September 2009 and December 2017. The index date was set as the date of questionnaire completion. Individuals younger than 18 years of age or older than 105 years of age were excluded. To create a primary prevention cohort, individuals who had a history of myocardial infarction, stroke, coronary revascularization, congestive heart failure, peripheral arterial disease (including carotid endarterectomy procedures or abdominal aortic aneurysms), and atrial fibrillation hospitalizations in the 20 years prior to entry in the cohort were excluded.^22^ Further exclusions included individuals with a history of metastatic cancer, moderate-severe liver disease, or dementia; those on chronic dialysis; and residents of long-term care facilities, since modification of many cardiovascular health factors (eg, cholesterol) and behaviors (eg, diet) may be less relevant or feasible in these patients.^24^^,^^25^

### Cardiovascular health

The criteria used to define ideal health behaviors and health factors are shown in Supplemental Table 1 and details are provided in the Supplemental Methods. The definitions for each metric were adapted from the CANHEART health index. This index was developed to examine the health of Canadians at the population level and has been subsequently used to assess health status in cohorts of patients with established chronic cardiovascular disease (CVD) conditions.^20^^,^^26^, ^27^, ^28^ The CANHEART health index originally included 6 health behaviors and factors (ie, body habitus, blood glucose, blood pressure, smoking, physical activity, and diet). In the present analysis, we not only included these 6 metrics but also an additional health behavior (ie, sleep duration) and factor (ie, blood cholesterol) concordant with the American Heart Association’s Life’s Essential 8. We utilized definitions tailored for self-reported data and considered broader definitions of ideal health that were recently developed by the American Heart Association.^2^^,^^21^^,^^29^, ^30^, ^31^, ^32^ The definitions for each factor accounted for the data availability in the OHS to improve generalizability to pan-Canadian cohorts that are currently recruiting participants in a similar fashion^23^ and future endeavors to apply the index to nationally representative samples in Canada. The presence of each health behavior or factor was assigned a value of 1 for an ideal state and 0 for a nonideal state. Then, to quantify overall health, all 8 values were summed to create the CANHEART health index (range: 0-8). Health categories were previously defined based on the association between the CANHEART health index and prevalent CVD in the Canadian population.^20^ We adopted similar categories of ideal (8 points), intermediate (5-7 points), and poor (0-4 points) for our analysis.

### Outcomes

The primary outcome of interest was a CVD event defined by cardiovascular death or hospitalization for myocardial infarction, angina, cerebrovascular events (ischemic stroke, hemorrhagic stroke, and transient ischemic attack), congestive heart failure, peripheral arterial disease (including abdominal aortic aneurysms and carotid endarterectomy or stenting) and coronary revascularization (percutaneous coronary intervention or coronary artery bypass grafting).^33^ Hospitalization outcomes were ascertained using previously validated algorithms.^22^^,^^34^, ^35^, ^36^ Cause-specific mortality outcomes were ascertained from the Ontario Registrar General Database and validated administrative algorithms.^37^ The secondary outcome was mortality from any cause. Patients were followed until the first of the outcomes of interest, mortality, or December 31st, 2023.

### Statistical analysis

Descriptive statistics (means, medians, and proportions) were estimated for demographic and clinical characteristics separately for women and men. The proportion of individuals achieving ideal health for each behavior or risk factor domain and overall cardiovascular health was reported after age-standardization to the 2016 Ontario population in women and men separately. The 95% CIs were estimated using the gamma distribution. The association between cardiovascular health status and each outcome was estimated using cause-specific hazards models (and a Cox model for all-cause mortality).^38^ In order to determine whether the association between cardiovascular health and outcomes differed in women and men, we fit a single model for both sexes and included an interaction term between sex and cardiovascular health status categories. Our primary analysis included individuals with nonmissing values for cardiovascular health status. All models were adjusted for age. Measures of association were reported as HRs along with 95% CIs. The proportional hazards assumption was verified through visual inspection of Schoenfeld residuals plotted against time. When violations were detected, interaction terms between the variables of interest and time were introduced into the regression model.

We performed a series of additional analyses to assess the robustness of our findings. First, we repeated the analysis after using multiple imputation for missing health variables.^39^ In this dataset, we also determined whether the observed sex differences in outcomes were attenuated after adjusting for additional clinical (obstructive sleep apnea, estimated glomerular filtration rate, and family history of CVD) and social determinants of health (self-reported race and ethnicity, level of education obtained, annual reported income, residence within geographic regions of Ontario that display variations in ambulatory care services and CVD incidence rates,^40^ residence in rural vs urban areas, recent immigration to Canada within the 20 years prior to index, and neighborhood-level income). Full details of the imputation methods are presented in the Supplemental Methods. Next, we repeated the analysis using Fine-Gray subdistribution hazards regression to ensure that the relative increase in rates observed with poor cardiovascular health was associated with increased incidences. Then, we restricted definitions of health variables to self-reported data alone to improve generalizability to future studies that may consider leveraging widely available Canadian survey data. Finally, for future studies in cohorts where a particular factor or behavior is not collected, and thus multiple imputation is not plausible, it would be important to know if health measured by 6 or 7 factors remains associated without outcomes and differs by sex. Hence, for individuals with 1 or 2 missing health variables in our cohort, we included them in the regression model by grading their health as ideal (6 points), intermediate (4-5 points), and poor (0-3 points) when 6 variables were present and ideal (7 points), intermediate (5-6 points), and poor (0-4 points) when 7 variables were present. Statistical analyses were conducted using SAS version 9.4 (SAS Institute).

## Results

### Study cohort

There were 183,545 individuals enrolled in OHS who completed the baseline questionnaire. There were 7,146 (3.9%) individuals with prior CVD, as well as 1,301 (0.7%) with severe comorbidities or residents of long-term care facilities that were excluded (Supplemental Figure 1). Baseline characteristics of the final cohort are presented in Table 1. The cohort consisted of 175,098 individuals, among whom 107,576 (61.4%) were women and 67,522 (38.6%) were men. The mean age in women was 45.8 years (SD: 13.9 years), while the mean age in men was 49.3 years (SD: 14.7 years).

**Table 1:** Baseline Characteristics of the Cohort

	Total (N = 175,098)	Female (n = 107,576)	Male (n = 67,522)
Demographics			
Age, y, mean (SD)	47.18 (14.33)	45.85 (13.93)	49.29 (14.69)
Traditional risk factors			
High blood cholesterol, n (%)	26,975 (20.3%)	12,331 (15.5%)	14,644 (27.7%)
Diabetes, n (%)	13,453 (7.7%)	6,557 (6.1%)	6,896 (10.2%)
Hypertension, n (%)	38,710 (22.1%)	20,481 (19.0%)	18,229 (27.0%)
Elevated waist-to-hip ratio, n (%)	58,143 (62.7%)	31,116 (57.7%)	27,027 (69.5%)
Total cholesterol ≥240 mg/dL, n (%)	6,636 (10.9%)	4,673 (13.2%)	1,963 (7.7%)
High-density lipoprotein cholesterol ≥60 mg/dL, n (%)	23,044 (38.0%)	18,026 (51.1%)	5,018 (19.8%)
Hemoglobin A1c ≥7%, n (%)	4,054 (12.9%)	1,861 (10.4%)	2,193 (16.3%)
Fasting glucose ≥7 mmol/L, n (%)	2,849 (7.5%)	1,201 (5.4%)	1,648 (10.4%)
Systolic blood pressure ≥140 mm Hg, n (%)	1,580 (13.4%)	685 (9.9%)	895 (18.3%)
Body mass index <25 kg/m^2^, n(%)	55,941 (41.1%)	38,207 (48.2%)	17,734 (31.2%)
Health behaviors			
Active daily smoker, n (%)	17,110 (10.5%)	10,934 (11.0%)	6,176 (9.8%)
5 or more servings/day of fruits and vegetables, n (%)	74,309 (46.5%)	52,822 (53.7%)	21,487 (34.9%)
Sleep duration of 7 to <9 hours/day, n (%)	99,993 (64.4%)	61,761 (64.8%)	38,232 (63.8%)
High physical activity level, n (%)	58,248 (38.2%)	33,850 (36.0%)	24,398 (41.6%)
Preventative treatment			
Antihypertensive therapy, n (%)	12,963 (7.4%)	6,361 (5.9%)	6,602 (9.8%)
Cholesterol-lowering therapy, n (%)	9,078 (5.2%)	3,450 (3.2%)	5,628 (8.3%)

Missing data are present for blood cholesterol (15.4%), waist-to-hip ratio (47%), total cholesterol (65.2%), high-density lipoprotein cholesterol (65.3%), hemoglobin A1C (82.1%), fasting glucose (78.3%), systolic blood pressure (93.3%), body mass index (22.3%), smoking (6.9%), fruit and vegetable consumption (9.5%), sleep duration (11.3%), and physical activity (12.8%).

### Cardiovascular health status

**Figure 1:**
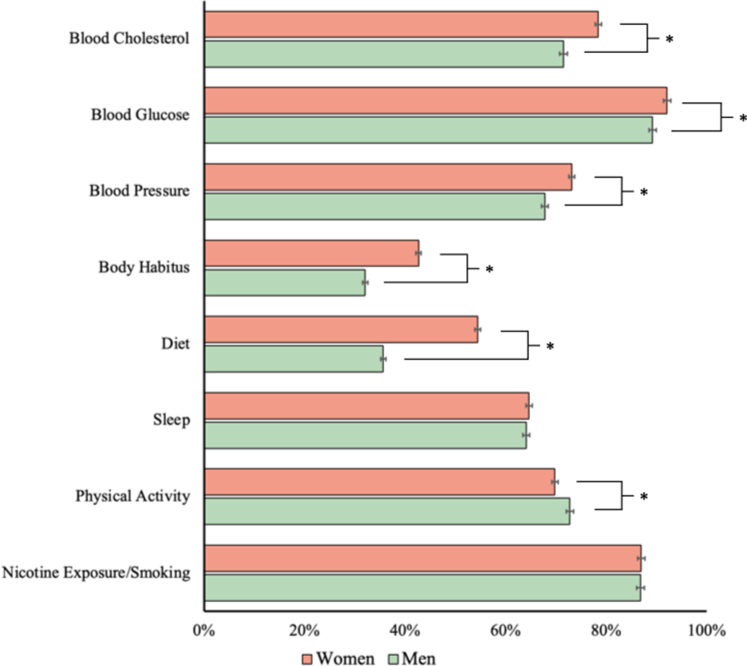
**Age-Standardized Prevalence of Ideal Cardiovascular Health Measures** The prevalence of each health factor is depicted for women and men after age standardization to the 2016 Ontario census. Bars and whiskers represent proportions with 95% CIs. Asterisks indicate nonoverlapping CIs.

Age-standardized baseline cardiovascular health is presented for women and men in Figure 1. Women were more likely to have ideal blood cholesterol (women: 78.4% vs men: 71.5%), blood glucose (92.1% vs 89.2%), blood pressure (73.2% vs 67.8%), and body habitus (42.7% vs 32.0%) in comparison to men. On the other hand, when considering health behaviors, women were less likely to have ideal physical activity levels (69.8% vs 72.8%) but more likely to have an ideal diet (54.4% vs 35.6%).

**Figure 2:**
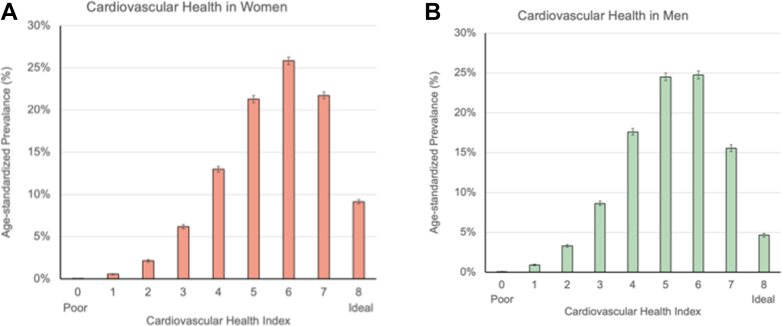
**Cardiovascular Health in Women and Men** The distribution of cardiovascular health is depicted for (A) women and (B) men after age-standardization to the 2016 Ontario census. Bars and whiskers represent proportions with 95% CIs.

The distribution of the overall cardiovascular health is presented in Figure 2 and ranged between 0 and 8 for both sexes. After age-standardization, the mean cardiovascular health index was 5.62 (95% CI: 5.60-5.65) in women and 5.21 (95% CI: 5.19-5.23) in men. There were more women compared to men with ideal cardiovascular health (women: 9.1% vs men: 4.6%) and fewer women with poor cardiovascular health (21.9% vs 30.5%).

### Cardiovascular health status and long-term cardiovascular outcomes

**Figure 3:**
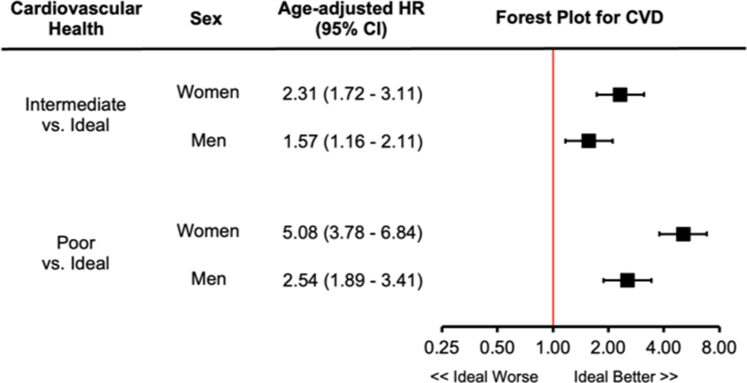
**The Association Between Cardiovascular Health and Cardiovascular Events** The association between the rate of cardiovascular events in women and men by poor (0-4 points), intermediate (5-7 points), and ideal (8 points) health status defined by the CANHEART Health Index is depicted. The model was adjusted for age. There existed a significant interaction between sex and health status for cardiovascular disease (*P* < 0.001). CVD = cardiovascular disease; CANHEART = Cardiovascular Health in Ambulatory Care Research Team.

After a median of 11.1 years of follow-up, the unadjusted rates of CVD for ideal, intermediate, and poor cardiovascular health in women were 0.7, 2.0, and 5.8 per 1,000 person-years, respectively. Compared to ideal cardiovascular health, the age-adjusted HR for intermediate cardiovascular health was 2.31 (95% CI: 1.72-3.11) and for poor cardiovascular health was 5.08 (95% CI: 3.78-6.84) in women. Similarly, in men, the unadjusted rate of CVD events increased for ideal, intermediate, and poor health from 2.4 to 5.5 to 12.6 per 1,000 person-years, respectively. Compared to ideal cardiovascular health, the age-adjusted HR for intermediate cardiovascular health was 1.57 (95% CI: 1.16-2.11), while the HR for poor cardiovascular health was 2.54 (95% CI: 1.89-3.41) (Figure 3). Interaction analysis demonstrated that the HRs were significantly larger for women compared to men for intermediate (HR: 2.31 vs 1.57) and poor health (HR: 5.08 vs 2.54; *P* < 0.001).

### Cardiovascular health status and all-cause mortality

**Figure 4:**
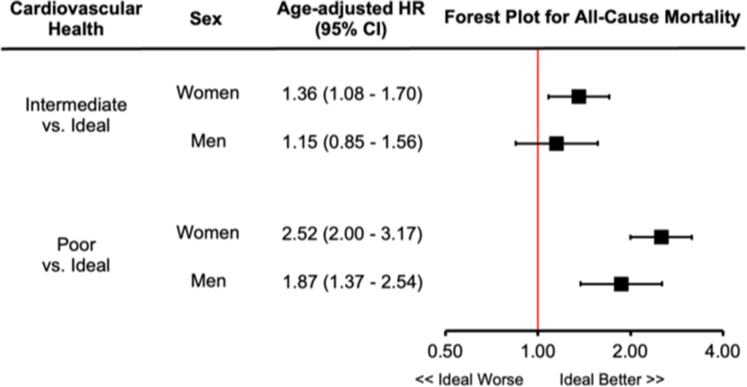
**The Association Between Cardiovascular Health and All-Cause Mortality** The association between the rate of all-cause mortality in women and men by poor (0-4 points), intermediate (5-7 points), and ideal (8 points) health status defined by the CANHEART Health Index is depicted. The model was adjusted for age. There existed a significant interaction between sex and health status for all-cause mortality (*P* = 0.042). CANHEART = Cardiovascular Health in Ambulatory Care Research Team.

In women, the rate of all-cause mortality increased from 1.2 to 2.1 to 5.4 per 1,000 person-years for ideal, intermediate, and poor health, respectively. Compared to ideal cardiovascular health, the age-adjusted HR was 1.36 (95% CI: 1.08-1.70) for intermediate cardiovascular health and 2.52 (95% CI: 2.00-3.17) for poor cardiovascular health. In men, the rate of all-cause mortality increased from 2.2 to 4.0 to 9.4 per 1,000 person-years. The corresponding age-adjusted HR was 1.15 (95% CI: 0.85-1.56) for intermediate cardiovascular health and 1.87 (95% CI: 1.37-2.54) for poor cardiovascular health (Figure 4). Sex modified the association between cardiovascular health and all-cause mortality such that the HR for women was larger than that of men for intermediate (HR: 1.36 vs 1.15) and poor cardiovascular health (HR: 2.52 vs 1.87; *P* = 0.042 for interaction).

### Additional and sensitivity analyses

For CVD but not death, minor violations of the proportionality assumption were detected for sex. When the effect of intermediate and poor health on CVD was modeled over time, results remained consistent such that the HR for women was larger than that of men throughout follow-up, although the overall effect attenuated over time (Supplemental Figure 2). Our findings remained consistent after multiple imputation for missing data (Supplemental Table 2). Cardiovascular health was associated with a higher rate of CVD and all-cause mortality over the follow-up period, and sex modified the interaction for each outcome (interaction *P* < 0.001 for CVD and all-cause mortality). Importantly, when adjusted for additional clinical and social determinants of health, the results remained similar, and the effect modification between sex and the cardiovascular health persisted for CVD (*P* < 0.001 for interaction) and all-cause mortality (*P* = 0.02). Results also remained consistent using Fine and Gray regression models (Supplemental Table 3) when restricting to data based on self-reported history of high blood pressure, cholesterol, or diabetes mellitus (Supplemental Table 4), as well as when grading individuals who had only 6 or 7 health variables available for analysis (Supplemental Table 5).

## Discussion

In this large, contemporary primary prevention cohort, we found that cardiovascular health was better in women compared to men, yet less than 1 in 10 women or men achieved ideal health. Cardiovascular health status was strongly associated with long-term outcomes. With declining cardiovascular health status, absolute rates of CVD were higher in men compared to women. However, the relative impact of cardiovascular health on future risk was higher for women than men. Poor health was associated with a 5-fold higher rate of CVD events in women and a 2.5-fold higher rate of CVD in men, highlighting the importance of considering sex-specific health promotion strategies (Central Illustration).

**Central Illustration:**
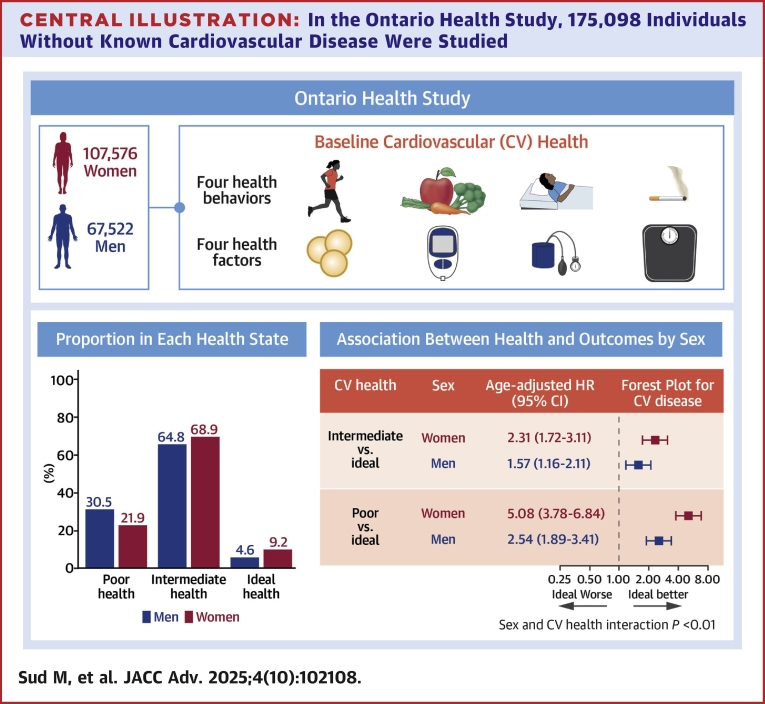
**In the Ontario Health Study, 175,098 Individuals Without Known Cardiovascular Disease Were Studied** Cardiovascular health was measured using 8 health factors and behaviors (diet, sleep, physical activity, smoking, body habitus, blood glucose, blood cholesterol, and blood pressure). Compared to ideal cardiovascular health, women had a greater age-adjusted HR for incident CVD when considering intermediate cardiovascular health (HR: 2.31 vs 1.57) or poor cardiovascular health (HR: 5.08 vs 2.54; *P* < 0.01 interaction). CVD = cardiovascular disease.

Optimizing health factors and behaviors remains the cornerstone of CVD prevention.^41^^,^^42^ Most studies have shown that few individuals have ideal cardiovascular health and more women than men have ideal cardiovascular health. For instance, studies from community cohorts in the United Kingdom^8^, China^6^, Europe^43^, and the United States^44^^,^^45^ showed that <5% of participants had ideal cardiovascular health. Furthermore, more women achieved ideal health for risk factors in comparison to men, yet fewer women achieved ideal health behaviors.^6^^,^^46^ In the Canadian Community Health Survey, 13% of women and 6% of men achieved ideal cardiovascular health without the inclusion of sleep or cholesterol.^20^ Our results were in keeping with the prior literature. We noted that only 9% of women and 5% of men had ideal cardiovascular health, and variations in ideal health existed for most factors between sexes. More women relative to men had ideal health factors (ie, blood glucose, blood cholesterol, blood pressure, and measures of body habitus). On the other hand, when considering health behaviors, more women displayed an ideal diet while fewer displayed ideal physical activity levels. These sobering findings highlight the worrisome overall health in Canadians and suggest that much work remains to be done to improve risk factor burden in women and men.

While numerous prior studies have demonstrated an association between ideal health and outcomes, it remains unclear whether the impact of health differs between women and men.^47^ For instance, sex did not modify the association between health and CVD outcomes in a community cohort from the United States.^46^ On the other hand, poorer health was associated with a larger impact on risk in women compared to men in the United Kingdom^8^ and Northern China.^6^ Our findings are in keeping with the latter two studies. Although absolute rates of CVD and mortality were lower in women, risk factors and behaviors were more important on a relative basis for women. For the same level of health, the relative rate of both CVD events and all-cause mortality was higher in women compared to men. It is recognized that differences in risk factor effects are likely a byproduct of biological and hormonal factors as well as social, cultural, and health system factors.^48^ Elucidating the contribution of each to the greater relative risk seen in women for each level of cardiovascular health was beyond the scope of this study. Nonetheless, we did note that our findings persisted despite adjustment for social determinants of health but we could not rule out differences in unmeasured sociocultural factors or risk factor management inequities during follow-up that could have contributed to the observed sex-specific impact on risk.^17^, ^18^, ^19^ Although our findings could be explained by differences in how ideal health was measured, it is still in keeping with extensive literature showing greater CVD risk in women for many health factors (eg, glucose^13^, blood pressure^11^) and behaviors (eg, physical activity^10^, smoking^9^, and sleep^12^^,^^49^). Hence, while improving population-level health should continue to remain a national (and global) priority, our study highlights the need to consider sex-specific interventions, education, and counseling to improve cardiovascular health and ensure equitable disease prevention among women and men.

### Study Limitations

A few limitations of our study are worth noting. First, health factors, behaviors, and anthropometric measurements are based, in part, on self-report from OHS. This may be subject to recall bias, resulting in a lower prevalence of reported risk factors. However, when possible, we supplemented self-report data with validated administrative algorithms for case definitions (eg, hypertension and diabetes mellitus) and provincial laboratory data (eg, cholesterol and hemoglobin A1C). Furthermore, there may also be social desirability bias such that individuals underreport factors related to obesity (height and weight) or low physical activity.^50^ While this could result in a lower prevalence of these 2 behaviors, it is likely to bias the association with outcomes toward the null. Nonetheless, future studies investigating cardiovascular health in Canada should consider standardized collection of health behaviors and factors to improve tracking population health. Second, while we were able to measure dietary patterns of fruits and vegetables, this does not take into account the full spectrum of dietary patterns that may contribute to cardiovascular health. Third, while our definitions using physical and lab measures align with disease diagnosis, lower values are generally considered more desirable for ideal health (eg, blood pressure <120 mm Hg vs <140 mm Hg). However, the current definitions used tend to bias our results toward, rather than away from, the null. Fourth, our data were subject to missingness, which may have biased the association between cardiovascular health and outcomes. However, multiple imputation suggested our results remained valid. Furthermore, even when one or two factors were not available, the association between cardiovascular health and outcomes persisted. Fifth, we did not have information on how cardiovascular health changed over time. It is conceivable that sex differences may exist in the trajectories of cardiovascular health that could explain the differences in baseline risk between women and men observed in our cohort and the attenuation in HRs between sexes over time. Finally, due to the voluntary enrollment into the OHS, there may be healthy participant bias, and thus, the study cohort may not be reflective of all Canadians. However, our findings that few participants achieved ideal health was in keeping with results from the Canadian Community Health Survey.

In conclusion, our findings suggest that poor health is common among middle-aged individuals without prior CVD. We have identified important prognostic differences between sexes that strengthen the rationale to consider sex-specific education and counseling to improve individual and population-level health. Our findings will help to refine future strategies to reduce CVD burden.

Perspectives **COMPETENCY IN MEDICAL KNOWLEDGE:** Our findings demonstrate that poor cardiovascular health, when measured with 4 health behaviors and 4 health factors, is common among middle-aged individuals without prior CVD. While more women compared to men have ideal cardiovascular health and fewer women have poor cardiovascular health, the relative impact of health on outcomes differs by sex. Poor health is associated with a higher rate of CVD of 5-fold in women and 2.5-fold in men.

**TRANSLATIONAL OUTLOOK:** The relative impact of cardiovascular health on outcomes differs by sex. Primary care teams can utilize this knowledge to approach prevention using sex-specific strategies, which can include individualized education and counseling to improve cardiovascular health measures.

## Funding support and author disclosures

Funding for this study was provided by a Foundation Grant (FDN 154333) and by the Heart & Stroke Polo Chair Early Career Investigator Award Funds. The funding source had no role in the study's design, conduct, or reporting. Dr Wijeysundera is supported by a 10.13039/501100001804Canada Research Chair in Structural Heart Disease Policy and Outcomes. Dr Madan is supported by the Heart and Stroke Foundation Polo Chair in Cardiology at the 10.13039/501100003579University of Toronto. Dr Ko is supported by the Jack Tu Chair in Cardiovascular Outcomes Research. This study was supported by ICES, which is funded by an annual grant from the 10.13039/501100000226Ontario Ministry of Health (MOH) and the Ministry of Long-Term Care (MLTC). The analyses, conclusions, opinions, and statements expressed herein are solely those of the authors and do not reflect those of the funding or data sources; no endorsement is intended or should be inferred. The views expressed therein are those of the author and do not necessarily reflect those of ORG, the Ministry of Public and Business Service Delivery, 10.13039/501100000008Health Canada, the 10.13039/100012118Ontario Institute for Cancer Research, or the 10.13039/100013873Government of Ontario. Parts or whole of this material are based on data and/or information compiled and provided by Immigration, Refugees and Citizenship Canada (IRCC) current to 2023. However, the analyses, conclusions, opinions and statements expressed in the material are those of the author(s), and not necessarily those of IRCC. Data contained herein were provided by the Ontario Health Study, which is supported by the Ontario Institute for Cancer Research through funding provided by the Government of Ontario. The authors have reported that they have no relationships relevant to the contents of this paper to disclose.
