# Supplementary material for: Sex Differences in Cardiovascular Health Status and Long-Term Outcomes in a Primary Prevention Cohort
Source: JACC Adv. 2025 Sep 1;4(10):102108. doi: 10.1016/j.jacadv.2025.102108 (PMC12421643; doi:10.1016/j.jacadv.2025.102108)
Supplement: Supplemental_Material [file mmc1.docx]

**SUPPLEMENTAL APPENDIX**

# Supplemental Methods for Defining Ideal Health

Ideal blood cholesterol was based on no self-reported history of high cholesterol diagnosed by a health care professional or recent non-high density lipoprotein cholesterol < 4.1mmol/L (160mg/dL). Ideal blood glucose was based on the absence of diabetes ascertained using validated administrative algorithms, self-report, hemoglobin A1C < 6.5% or fasting serum glucose < 7mmol/L (1). Ideal blood pressure was based on no previous hypertension diagnosis through administrative linkages, self-report, anti-hypertensive medication use, or a systolic blood pressure < 140 mmHg for individuals who attended in-person physical assessments as part of the Ontario Health Study (2,3). Ideal body habitus was based on body mass index < 25 kg/m^2^ estimated from self-reported weight and height, or if not available, self-reported waist-to-hip ratio (< 0.9 in men and <0.85 in women). Ideal fruit and vegetable consumption was based an average of 5 or more fruits or vegetables consumed per day based on previous studies demonstrating prognostic importance (4). Ideal physical activity thresholds were graded as moderate or high using the International Physical Activity Questionnaire (IPAQ) administered as part of the baseline study questionnaire (5). Ideal sleep duration was based on 7 to less than 9 self-reported hours of sleep on average per day (6). Ideal smoking was considered as no self-reported history of current cigarette use.

# Supplemental Methods for Multiple Imputation

Multiple imputation was performed for missing CANHEART variables (7). We elected to use a passive imputation approach for derived variables. A total of 36 multiply imputes datasets were generated based on the variable with the largest proportion of missing data. In addition to the age and the CANHEART variables, status indicators as well as crude hazard rates for the time-to-event outcome of CVD and death were included in the imputation model. Additional clinical factors (obstructive sleep apnea, estimate glomerular filtration rate, family history of cardiovascular disease) and social determinants of health (self-reported race and ethnicity, level of education obtained, annual reported income, residence within one of three geographic regions in Ontario defined by Local Health Integration Networks that are associated with ambulatory care service utilization and CVD incidence rates (8), residence in rural vs. urban areas, neighbourhood level income, and immigration to Canada within the last 20 years) were included in the imputation model that had missing values at baseline. In each multiply imputed dataset, the time-to-event analyses was repeated, and results were pooled using Rubin’s rule. The observed sex-differences in outcomes were also determined after adjusting for the additional multiply imputed clinical factors and social determinants of health.

# Supplemental Figure 1. Study Cohort Creation.


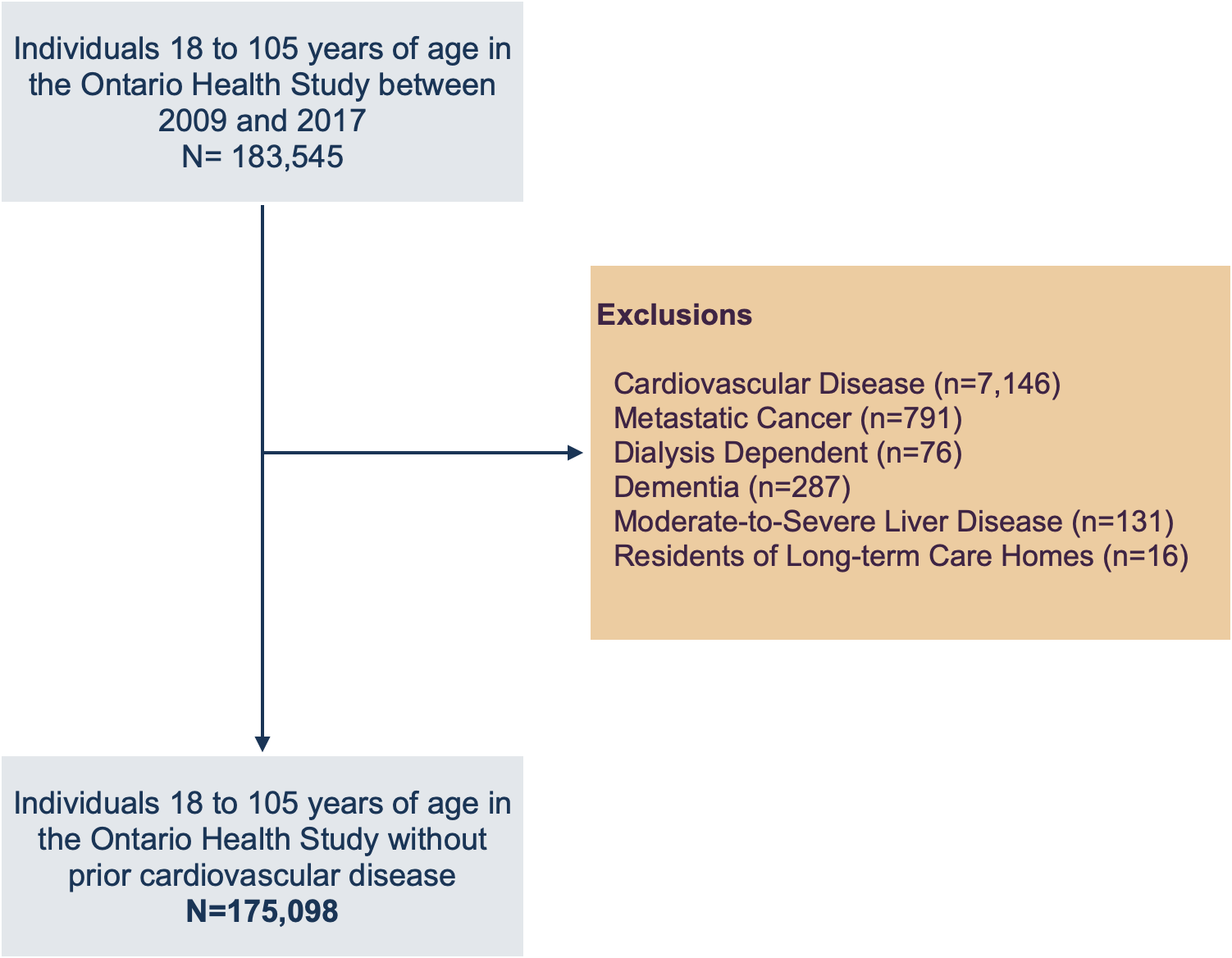


# Supplemental Figure 2: Association between CANHEART Health Index and Cardiovascular Disease Over Time. The association between the rate of cardiovascular events in women and men by poor (0-4 points), intermediate (5-7 points) and ideal (8 points) health status defined by the CANHEART Health Index is depicted. The model was adjusted for age. An interaction between sex, the CANHEART Health index and time was introduced into the model. CVD – cardiovascular disease.

# Supplemental Table 1. Definitions of Ideal Cardiovascular Health States in the CANHEART Health Index

| **Measure** | **Definition for Ideal Health^*^** |
| --- | --- |
| **Health Factors** | |
| Blood Cholesterol | No history of high cholesterol^†^ |
|  | Non-high-density lipoprotein < 4.1 mmol/L (160 mg/dL) |
| Blood Glucose | No history of diabetes mellitus^‡^ |
|  | Hemoglobin A1c < 6.5% |
|  | Fasting glucose < 7.0mmol/L |
| Blood Pressure | No history of hypertension^‡^ |
|  | Systolic blood pressure < 140 mmHg |
|  | Not currently using anti-hypertensive medication^†^ |
| Body Habitus | Waist-to-hip ratio < 0.9 in men / < 0.85 in women^†^ |
|  | Body Mass Index < 25 kg/m^2†^ |
| **Health Behaviours** | |
| Diet | ≥ 5 combined servings of fruit and vegetables per day^†^ |
| Sleep | 7 to < 9 hours of sleep per day^†^ |
| Physical activity | Moderate-to-high physical activity levels based on the IPAQ score^†^ |
| Smoking | Not currently smoking cigarettes^†^ |

^*^ – Ideal health defined when one of the listed criteria are met

^†^ – based on self-reported history

^‡^ – based on self-reported history or administrative linkage

IPAQ: International Physical Activity Questionnaire

# Supplemental Table 2. The Association Between the CANHEART Health Index and Outcomes after Multiple Imputation

| **Sex** | **Outcome** | **CANHEART Health Index** | **Hazard Ratio (95% CI)*** | | |
| --- | --- | --- | --- | --- | --- |
|  |  |  | **Unadjusted** | **Age-Adjusted** | **Fully Adjusted** |
|  |  |  |  |  |  |
| Female | Cardiovascular Disease | Ideal | **Reference** | | |
|  |  | Intermediate | 2.96 (2.22 - 3.93) | 2.43 (1.83 - 3.23) | 2.24 (1.68 - 2.97) |
|  |  | Poor | 9.81 (7.36 - 13.08) | 5.75 (4.31 - 7.65) | 4.61 (3.46 - 6.15) |
|  | All-Cause Mortality | Ideal | **Reference** | | |
|  |  | Intermediate | 1.98 (1.61 - 2.44) | 1.57 (1.28 - 1.93) | 1.44 (1.17 - 1.77) |
|  |  | Poor | 5.82 (4.73 - 7.15) | 3.15 (2.56 - 3.87) | 2.49 (2.02 - 3.07) |
|  |  |  |  |  |  |
| Male | Cardiovascular Disease | Ideal | **Reference** | | |
|  |  | Intermediate | 2.21 (1.69 - 2.88) | 1.57 (1.21 - 2.05) | 1.45 (1.12 - 1.89) |
|  |  | Poor | 5.60 (4.28 - 7.31) | 2.66 (2.04 - 3.47) | 2.21 (1.69 - 2.88) |
|  | All-Cause Mortality | Ideal | **Reference** | | |
|  |  | Intermediate | 2.10 (1.56 - 2.84) | 1.44 (1.07 - 1.93) | 1.34 (0.99 - 1.80) |
|  |  | Poor | 5.49 (4.08 - 7.39) | 2.42 (1.81 - 3.25) | 2.04 (1.52 - 2.74) |
|  |  |  |  |  |  |

* After multiple imputation in 36 data sets. In each model, the interaction between sex and the CANHEART Health Index was p < 0.05. Fully adjusted model included clinical (obstructive sleep apnea, estimate glomerular filtration rate, family history of cardiovascular disease) and social determinants of health (self-reported race and ethnicity, level of education obtained, annual reported income, previously defined geographic regions of residence associated with cardiovascular disease, residence in rural vs. urban areas, neighbourhood level income, and immigration status) as covariates. CI: confidence interval

# Supplemental Table 3. The Association Between the CANHEART Health Index and the Incidence of Outcomes

| **Sex** | **Outcome** | **CANHEART Health Index** | **Sub-distribution Hazard Ratio (95% CI)*** |
| --- | --- | --- | --- |
|  |  |  | **Age-Adjusted** |
|  |  |  |  |
| Female | Cardiovascular Disease | Ideal | **Reference** |
|  |  | Intermediate | 2.32 (1.73 - 3.11) |
|  |  | Poor | 5.05 (3.75 - 6.78) |
|  |  |  |  |
| Male | Cardiovascular Disease | Ideal | **Reference** |
|  |  | Intermediate | 1.57 (1.17 - 2.11) |
|  |  | Poor | 2.49 (1.86 - 3.35) |
|  |  |  |  |

* The interaction between sex and the CANHEART Health Index in Fine-Gray sub-distribution hazards models was p < 0.01. CI: confidence interval

# Supplemental Table 4. The Association Between the CANHEART Health Index and Outcomes Using Self-Report Survey Data

| **Sex** | **Outcome** | **CANHEART Health Index** | **Hazard Ratio (95% CI)*** |
| --- | --- | --- | --- |
|  |  |  | **Age-Adjusted** |
|  |  |  |  |
| Female | Cardiovascular Disease | Ideal | **Reference** |
|  |  | Intermediate | 2.38 (1.77 - 3.22) |
|  |  | Poor | 5.38 (3.97 - 7.28) |
|  | All-Cause Mortality | Ideal | **Reference** |
|  |  | Intermediate | 1.37 (1.09 - 1.72) |
|  |  | Poor | 2.71 (2.15 - 3.43) |
|  |  |  |  |
| Male | Cardiovascular Disease | Ideal | **Reference** |
|  |  | Intermediate | 1.54 (1.16 - 2.03) |
|  |  | Poor | 2.45 (1.86 - 3.25) |
|  | All-Cause Mortality | Ideal | **Reference** |
|  |  | Intermediate | 1.29 (0.94 - 1.76) |
|  |  | Poor | 2.16 (1.58 - 2.96) |
|  |  |  |  |

* The interaction between sex and the CANHEART Health Index was p < 0.01 for cardiovascular disease and p = 0.04 for all-cause mortality. CI: confidence interval

# Supplemental Table 5. The Association Between the CANHEART Health Index and Outcomes when Six or More Variables are Available

| **Sex** | **Outcome** | **CANHEART Health Index*** | **Hazard Ratio (95% CI)**** |
| --- | --- | --- | --- |
|  |  |  | **Age-Adjusted** |
|  |  |  |  |
| Female | Cardiovascular Disease | Ideal | **Reference** |
|  |  | Intermediate | 2.08 (1.70 - 2.55) |
|  |  | Poor | 4.82 (3.93 - 5.91) |
|  | All-Cause Mortality | Ideal | **Reference** |
|  |  | Intermediate | 1.36 (1.16 - 1.61) |
|  |  | Poor | 2.72 (2.31 - 3.21) |
|  |  |  |  |
| Male | Cardiovascular Disease | Ideal | **Reference** |
|  |  | Intermediate | 1.68 (1.35 - 2.10) |
|  |  | Poor | 2.78 (2.23 - 3.47) |
|  | All-Cause Mortality | Ideal | **Reference** |
|  |  | Intermediate | 1.37 (1.08 - 1.74) |
|  |  | Poor | 2.31 (1.82 - 2.92) |
|  |  |  |  |

* For individuals who had one missing CANHEART variables, health was graded poor, intermediate, and ideal based on 0 – 3, 4 – 5, and 6 points, respectively. For those who had two missing variables, health was graded as poor, intermediate, and ideal based on 0 – 4, 5 – 6, and 7 points, respectively.

** The interaction between sex and the CANHEART Health Index was p < 0.01 for cardiovascular disease and p < 0.01 for all-cause mortality. CI: confidence interval

# References

1. Lipscombe LL, Hwee J, Webster L, Shah BR, Booth GL, Tu K. Identifying diabetes cases from administrative data: a population-based validation study. BMC Health Serv Res 2018;18:316.

2. Kirsh VA, Skead K, McDonald K et al. Cohort Profile: The Ontario Health Study (OHS). Int J Epidemiol 2022.

3. Tu K, Campbell NR, Chen ZL, Cauch-Dudek KJ, McAlister FA. Accuracy of administrative databases in identifying patients with hypertension. Open Med 2007;1:e18-26.

4. Wang DD, Li Y, Bhupathiraju SN et al. Fruit and Vegetable Intake and Mortality: Results From 2 Prospective Cohort Studies of US Men and Women and a Meta-Analysis of 26 Cohort Studies. Circulation 2021;143:1642-1654.

5. Sjostrom M, Ainsworth BE, Bauman A, Bull FC, Hamilton-Craig CR, Sallis JF. Guidelines for data processing analysis of the International Physical Activity Questionnaire (IPAQ) - Short and long forms. 2005.

6. Makarem N, Castro-Diehl C, St-Onge MP et al. Redefining Cardiovascular Health to Include Sleep: Prospective Associations With Cardiovascular Disease in the MESA Sleep Study. J Am Heart Assoc 2022;11:e025252.

7. Austin PC, White IR, Lee DS, van Buuren S. Missing Data in Clinical Research: A Tutorial on Multiple Imputation. Can J Cardiol 2021;37:1322-1331.

8. Tu JV, Chu A, Maclagan L et al. Regional variations in ambulatory care and incidence of cardiovascular events. CMAJ 2017;189:E494-E501.
